# Evaluation of Mutton Quality Characteristics of Dongxiang Tribute Sheep Based on Membership Function and Gas Chromatography and Ion Mobility Spectrometry

**DOI:** 10.3389/fnut.2022.852399

**Published:** 2022-05-06

**Authors:** Zengkui Lu, Jianye Li, Chao Yuan, Bin Xi, Bohui Yang, Xianyu Meng, Tingting Guo, Yaojing Yue, Yaqin Gao, Jianbin Liu, Xiaoping Sun

**Affiliations:** ^1^Lanzhou Institute of Husbandry and Pharmaceutical Sciences, Chinese Academy of Agricultural Sciences, Lanzhou, China; ^2^Sheep Breeding Engineering Technology Research Center of Chinese Academy of Agricultural Sciences, Lanzhou, China; ^3^Quality Safety Risk Assessment of Animal Products, Ministry of Agriculture, Lanzhou, China; ^4^Dongxiang Autonomous County Animal Husbandry Development Center, Linxia, China

**Keywords:** dongxiang tribute sheep, membership function, GC-IMS, quality characteristic, volatile compound

## Abstract

Dongxiang tribute sheep have a history of use in food dishes such as “Dongxiang Handgrip,” which dates back hundreds of years and is a favorite halal food in northwestern China. However, little is known about the mutton quality characteristics of Dongxiang tribute sheep. Here, we measured the sensory characteristics, nutritional quality, and flavor substances to comprehensively evaluate the mutton quality characteristics of these sheep. The mutton qualities of Dongxiang tribute, Tibetan, Ujumqin, and Hu sheep were comprehensively evaluated by membership function. Subsequently, the volatile components in mutton samples from 30 Dongxiang tribute sheep were detected *via* gas chromatography and ion mobility spectrometry (GC-IMS), and their fingerprints were established. The result of meat quality revealed that the shear force, the contents of protein, essential amino acid (EAA), non-essential amino acid (NEAA), and n-6/n-3 ratio of Dongxiang tribute mutton were better than the other three breeds. Membership functions were calculated for 10 physical and chemical indexes of mutton quality, and the comprehensive membership function values of the four breeds in order of highest to lowest mutton quality were Tibetan sheep (0.76) > Dongxiang tribute sheep (0.49) > Hu sheep (0.46) > Ujumqin sheep (0.33). Thirty volatile compounds were identified *via* GC-IMS: seven alcohols, eight aldehydes, five ketones, two esters, two phenols, one ether, one furan, one acid, two hydrocarbons, and one pyrazine. Ketones, aldehydes, and alcohols were the main volatile compounds forming the flavor of Dongxiang tribute sheep mutton. The reliability of the results was validated by PCA (principal component analysis) and similarity analyses. Our results provide reference value for consumers of mutton in China.

## Introduction

Dongxiang tribute sheep are an exotic breed that was transferred into China with Genghis Khan’s eastern expedition during the 10th century and have been highly regarded throughout history. A record of “87 tribute sheep soup” was noted in the annals of Hezhou in as early as the Ming Dynasty. Dongxiang tribute sheep are produced in Dongxiang Autonomous County of Linxia Hui Autonomous Prefecture of Gansu Province and its adjacent areas. The special arid geographical environment of the Dongxiang area led to the breed’s good mutton quality and to it being considered a superior tribute in ancient times; therefore, the sheep were named “Dongxiang tribute sheep.” Importantly, Dongxiang tribute sheep have been approved by the state trademark administration and have become a national geographical indication product. However, reports on the quality characteristics of Dongxiang tribute sheep mutton are limited.

Consumers favor mutton for its taste, tenderness, high nutritional value, and low cholesterol; however, some consumers dislike its unique flavor (1). Flavor is the sensory impression of a food and is the dominant factor determining the mutton quality and consumers’ purchasing decisions (2). When making purchasing decisions, consumers can evaluate mutton quality only through vision and smell, but evaluating it by flavor is difficult. Many factors, such as breed, age, sex, feeding method, slaughtering method, and processing procedure, affect the mutton’s flavor (3–5). Flavor generally includes taste and aroma. Taste comes from organic acids, inorganic salts, inosinic acid, free amino acids, and ribose, among which, inosinic acid is the key compound determining the freshness of the mutton (6, 7). Aroma is the reduction and decomposition of flavor precursors during cooking, including volatile compounds such as sulfides, unsaturated aldehydes, acids, and heterocyclic compounds. Mutton is traditionally cooked in most areas of China, and the environmental conditions cause variations in the mutton flavor. Flavor substances vary widely and have complex components, small contents, and remarkable effects. Therefore, scholars should study how flavor substances form to produce high-quality mutton.

Many factors affect mutton quality, but the advantages and disadvantages of these factors are often subjective, which makes quantifiable evaluation of the quality difficult. Fuzzy mathematics enables making these calculations because the constitution of quality factors is pluralistic, imbalanced, and fuzzy. In fuzzy mathematics, the membership function is an indicator for comprehensively evaluating different memberships and is often used in researching quality and stress resistance of crops such as fruits, vegetables and forage. As the membership function is considered a reliable evaluation method (8–11), its use in evaluation livestock and poultry meat quality is also a feasible. Gas chromatography and ion mobility spectrometry (GC-IMS) is a recently developed separation and detection technology that enables quickly detecting volatile flavor compounds (12). Because of its advantages of high sensitivity, fast detection and simple operation, GC-IMS has been widely applied to identify food authenticity, evaluate quality, and detect flavor (12–15).

Here, we determined the quality characteristics of Dongxiang tribute sheep mutton and combined the existing quality data on Tibetan, Ujumqin, and Hu sheep to comprehensively analyze the quality of Dongxiang tribute mutton by membership function. We also established the fingerprints of the volatile compounds in Dongxiang tribute mutton *via* GC-IMS technology. These results will contribute to the brand building of Dongxiang tribute sheep.

## Materials and Methods

### Habitat Environment of Dongxiang Tribute Sheep

Dongxiang Autonomous County lies on the southwest portion of central Gansu Province, Eastern Linxia Hui Autonomous Prefecture, between 103°10′–44′ east longitude and 35°30′–36′ north latitude and has a temperate, semi-arid, continental climate. As the transition zone between the Loess Plateau and Qinghai-Tibet Plateau, the topography of the Dongxiang Autonomous County region is complex, with undulating mountains, vertical and horizontal gullies, and varying altitudes (1,736–2,664 m), with an average elevation of 2,199.5 m. Additionally, the Yellow, Taohe, and Daxia Rivers flow through the territory with good water quality and low mineralization. The annual average temperature is 5–9°C; the annual daylight hours are 2,497 h, the annual average precipitation is 535.00 mm, the annual evaporation reaches 1,437 mm, and the frost-free period is 138 days. It is hot at noon in the summer, cool in the mornings and evenings, and the longer winter period has large temperature differences between day and night. The average soil organic matter content is 3.14%, and the average pH is 6.48. The area is dominated by chestnut soil, chernozem, and lime soil and has good soil quality, with >8,000 types of natural vegetation. The vegetation mainly includes Gramineae, Leguminosae, Compositae, Salicaceae, Chenopodiaceae, and Liliaceae, and the hay is alkaline. Artificially planted forages mainly include alfalfa, green oats, and silage corn. As of the end of 2020, the county has raised 1.54 million sheep, of which 6,80,000 are in stock and 8,60,000 have been sold.

### Sample Collection

From within the borders of Dongxiang Autonomous County, we selected 30 half-house-feeding, full-grazing, 6-month-old Dongxiang tribute sheep (ram) and euthanized them *via* phenobarbital (Fatal-Plus, 10 mg/kg body weight, Vortech Pharmaceuticals, MI, United States) injection. We then collected 1,000 g of the *longissimus dorsi* muscle, which was cut into small pieces, transported back to the laboratory in fresh-keeping bags at 4°C, and stored at −80°C.

### Determination of Mutton Quality Indexes

Meat color: during 30 min after slaughtering, the probe of color different meter was placed vertically on the cross section of the sample after correction, and the values of brightness (L*), redness (a*), and yellowness (b*) were measured and three biological replicates were performed.

pH value: when 45 min was slaughtered, the pH value was measured by inserting the probe into the meat sample after the acidity meter was calibrated and repeated three times.

Water loss rate: the meat sample was cut into a cuboid with a diameter 5 cm and a thickness 1 cm, which was weighed as m_1_. Then, put 16 layers of neutral filter paper on the top and bottom of the sample, finally put 35 kg pressure on the tester platform and maintain 5 min, and immediately weigh the sample and record it as m_2_. *Water loss rate* = (m_1_−m_2_)/m_1_×100%, each repeated three times.

Cooked meat percentage: a total of 80 g sample was weighed (m_1_), heated for 30 min in 80°C water bath pot (in plastic bags) and then cooled to room temperature, the excess water on the sample surface was absorbed by the filter paper and weighed it (m_2_). *Cooked meat percentage* = m_2_/m_1_ × 100%, repeated three times.

Moisture content was determined *via* the direct drying method as per the GB 5009.3-2016 National Food Safety Standard “Determination of moisture in food” (16); Protein content was determined *via* the semi-trace Kjeldahl nitrogen determination method (the protein conversion factor is 6.25) as per the GB 5009.5-2016 National Food Safety Standard “Determination of protein in food” (17); Fat content was determined *via* the Soxhlet fat extractor method as per the GB 5009.6-2016 National Food Safety Standard “Determination of fat in food” (18); Ash content was determined *via* the burning method as per the GB 5009.7-2016 National Food Safety Standard “Determination of ash in food” (19).

Amino acid was determined according to GB 5009.124-2016 National Food Safety Standard “Determination of amino acid in food,” using an amino acid analyzer (20), with a ≥0.99 linearity of amino acid determination, 98–102% recovery rate, and ≤6% relative standard deviation.

Fatty acids were determined according to GB 5009.168-2016 National Food Safety Standard “Determination of fatty acid in food,” using an internal standard method (21), with a ≥0.99 linearity of amino acid determination, 80–110% recovery rate, and ≤4% relative standard deviation.

### Calculation of the Comprehensive Membership Function

To evaluate the Dongxiang tribute mutton quality by comprehensive membership function, we measured the physical and chemical indexes of the mutton quality of these sheep, combined with previously published mutton quality data of Tibetan, Ujumqin, and Hu sheep (22). Briefly, the positive index membership function value was calculated using the formula, *Xij*(u) = (*Xij*−*Xjmin*)/(*Xjmax*−*Xjmin*); and the negative index membership function value was calculated using the formula, *Xij*(u) = 1−(*Xij*−*Xjmin*)/(*Xjmax*−*Xjmin*). Among them, Xij(u) is the membership function value of the j-th index of the i-th breed, Xij is the measured value of the j-th index of the i-th breed, Xjmin is the minimum value of the j-th index in the tested breed, and Xjmin is the maximum value of the j-th index among the tested breed. The value of the comprehensive membership function was calculated as $\text{Xi}\left(\text{u}\right)=\left[\sum_{\text{i}=1}^{\text{n}}\text{Xij}\left(\text{u}\right)\right]÷\text{n}$ (8, 11).

### Constructing Fingerprints of the Volatile Flavor Substances

Gas chromatography and ion mobility spectrometry (Flavorspec, G.A.S. Instrument, Germany) and an SE-54 capillary column (Restek, United States) were used to analyze the volatile compounds in the Dongxiang tribute mutton. After thawing the samples for 12 h at 4°C, 3.0 g of each sample was accurately weighed and incubated for 15 min at 80°C in a 20-ml headspace bottle with a magnetic screw sealing cap. Subsequently, 500-ul samples were injected in splitless mode by injection needle under the following conditions: chromatographic column temperature: 60°C, drift tube temperature: 60°C, drift gas flow: 150 ml/min (constant), and nitrogen carrier gas purity: 99.999%. The flow rate of the GC chromatographic column was set at 2 ml/min for 2 min, then 20 ml/min for 8 min, then 100 ml/min for 15 min, then immediately stopped. N-ketone C4-C9 was used as the external standard to calculate the retention index (RI). The volatile compounds were identified by comparing the drift times of the standards in the RI and GC-IMS libraries. Volatile flavor substances were analyzed using Laboratory Analytical Viewer (LAV) software and NIST and IMS, and flavor substances were qualitatively analyzed in the NIST and IMS databases built in the GC-IMS Library Search software. Difference maps and fingerprints of the volatile organic compounds were constructed using the Reporter and Gallery plug-ins in LAV. The principal component analysis (PCA) was performed using Dynamic PCA plug-ins, and similarity analysis was performed using a matching matrix plug-in. Linearity of GC-IMS determination method ≥0.99, recovery ≥95%, relative standard deviation ≤5%.

The three-dimensional (3D) spectrum is shown with a blue background; the X-axis represents the ion migration time after normalization; the Y-axis represents the GC retention period, and the Z-axis represents the RIP (reactive ion peak) after normalization. Each point on either side of the RIP represents a volatile organic compound. White and red indicate lower and higher concentrations, respectively; the darker color indicates a greater concentration. The two-dimensional (2D) map uses the topographic map of sample No. 1 as a reference (blue background) and derives the topographic map for samples No. 2 to 30. If the volatile compounds in two samples are the same, the background of deduction is white; red indicates a higher volatile compound concentration than the reference, and blue indicates a lower volatile compound concentration. In the fingerprint, each row represents a sample, each column represents a volatile compound, and the color represents the content of the volatile compounds, with a brighter color indicating a higher content.

### Statistical Analysis

All data were represented by mean ± SD, and statistical analysis was performed using one-way analysis of variance by SPSS software, the *p-*values <0.05 was considered statistically significant.

## Results and Discussion

### Screening of the Mutton Quality Index to Calculate Comprehensive Membership Function Values

The pH of the mutton decreased from 7 to 5.3–5.8 at 24 h after slaughter, which is related to the rate of muscle glycogen glycolysis and pre-slaughter stress (23, 24). An abnormal pH affects meat color, shear force and the cooked meat rate. The pH of the mutton for all four breeds ranged from 6.31 to 6.83 (Supplementary Table 1), which is within the range reported in previous studies (23, 24). Meat color directly determines consumers’ purchasing inclination, and the myoglobin and hemoglobin contents directly affect the meat color. When red meat is bright cherry red, consumers typically regard it as high-quality (25, 26). Additionally, consumers believe that fresh mutton with an a* value >9.5 is acceptable (27). In this study, the L* and b* values of the four breeds did not differ significant; however, the a* value (10.14) of Dongxiang tribute mutton was extremely significantly lower than that of Tibetan, Ujumqin and Hu sheep (Supplementary Table 1), which may have been caused by dietary differences (28). The cooked meat rate is an index to measure cooking loss, and a higher cooked meat rate indicates better water retention and a higher cooked meat yield. The cooked meat rate of the Dongxiang tribute sheep was significantly higher than that of the Ujumqin sheep but lower than that of the Tibetan sheep (Supplementary Table 1). The water loss rate was strongly correlated with meat color, tenderness, and juiciness. A lower water loss rate typically results in better water retention, softer meat and higher quality. The water loss rate of the Dongxiang tribute sheep was extremely significantly higher than that of the other three breeds, indicating that Dongxiang tribute mutton is tougher (Supplementary Table 1). Corresponding changes in muscle structure and composition occur after slaughter, including glycolysis, protein degradation, and a reduced pH, all of which affect the water loss rate of the meat (29). Additionally, a greater shear force directly results in reduced tenderness. Shear force did not significantly differ among the four breeds, but the shear force of the Dongxiang tribute sheep was the lowest (Supplementary Table 1). A lower shearing force typically results in more tender meat; thus Dongxiang tribute mutton is the most tender. When the pH and meat color are within the normal range, the influence on meat quality is small and difficult to quantify. Thus, of these physical indexes, we used cooked meat rate, water loss rate and shear force to calculate membership function.

Moisture, protein, fat, and ash content are important indicators for evaluating the nutritional value of muscles. Moisture content is directly related to the mutton color and tenderness. Generally, when the moisture content is approximately 70%, the meat is fatter, and the moisture content is lower (30). The moisture content of the four mutton breeds was approximately 75%, which is in the normal range (Supplementary Table 2). Tibetan sheep are primarily graziers and thus have a higher lean meat rate and the lowest moisture content. Protein is an important component of tissues and organs, is the direct carrier of human and animal life activities, and plays a vital role in human growth and development (31). As a good source of dietary protein, 100 g of lean mutton contains approximately 20 g protein. Of the four breeds, Dongxiang tribute mutton had the highest protein content, conferring a potentially high-quality meat (Supplementary Table 2). Protein content can also be related to the rate of protein synthesis in the muscle. Fat content directly affects the tenderness, juiciness and flavor of meat; however, too much fat can affect consumers’ purchasing decisions. Among the four breeds of mutton, Tibetan sheep had the lowest fat content, and Hu sheep had the highest fat content; this may be related to their feeding methods (Supplementary Table 2). The fat content of lambs fed concentrated feed (house feeding) was higher than that of lambs fed forage (grazing) (32, 33). As consumers’ quality of life improves, they are increasingly looking for low-fat food. Although fat content directly affects meat quality, consumers still tend to prefer low-fat meat (34, 35). Although fat content directly affects meat quality, consumers still tend to prefer low-fat meat. Generally, consumers can accept a mutton fat content below 5% (36). The fat content of the four mutton breeds in this study were <5%, which meets low-fat requirements. Ash content is the basis for evaluating the mineral contents in food, and Dongxiang tribute mutton had the lowest ash content, indicating that this mutton contains is low in minerals (Supplementary Table 2). Based on these results, we selected protein, fat, and ash from the conventional nutritional components to calculate membership function.

Amino acid composition is a major indicator of meat protein nutrition and an important factor affecting meat quality (37). A compositional proportion of essential amino acids closer to that of human amino acids indicates a better quality protein, which higher absorption, utilization and application values (38). The essential amino acid (EAA) and non-essential amino acid (NEAA) requirements for adult men are 0.18 and 0.48 g/kg per day, respectively, with a required ratio of EAA/NEAA = 37.5% (39). Here, the EAA/NEAA of the four mutton breeds ranged from 64.81 to 70.72, which far exceeds the recommendations of the Food and Agriculture Organization, the World Health Organization and the United Nations University (Supplementary Table 3). These data suggest that mutton is an excellent protein source. The EAA and NEAA contents were highest in the Dongxiang tribute sheep mutton and corresponded to the protein contents of conventional nutrients (Supplementary Table 3). Furthermore, amino acids in meat can interact with each other to contribute to aroma production during cooking. For example, aspartic acid and glutamic acid are known as umami amino acids, and their higher contents contribute to the richer taste of mutton. Consistent with other studies, glutamate had the highest content among all amino acids in all four mutton breeds (40, 41). Leucine is important for aroma formation because it provides Strecker aldehydes (42). Leucine was highest in the Tibetan mutton and lowest in the Dongxiang tribute mutton. Alanine and arginine can also improve the aroma of meat, with the highest contents in Dongxiang tribute mutton (5). In conclusion, the differences in amino acid compositions and contents among the four breeds were small, which was consistent with other studies that found that the amino acid compositions in meat were minimally affected by feed or breed (5, 43). Thus, we selected EAA/NEAA and umami amino acid content for membership function calculation.

The fatty acid composition in meat is important because it is inextricably linked with human health (44, 45). Similarly, the fatty acid composition affects meat quality parameters, such as juiciness, flavor, and shelf-life (5, 46). Sheep fat is similar to fat from other ruminants such as cattle. Consistent with our results, the fats in ruminant sediments include mainly saturated and monounsaturated fats, and C16:0, C18:0, and C18:1 c9 account for approximately 80% of the total fatty acids (47). Studies have shown that an increased C18:0 content makes mutton taste heavier (goaty), and the Dongxiang tribute mutton had the lowest C18:0 content (48). Studies have shown that excessive intake of saturated fatty acids (SFA) is a high risk factor for cardiovascular diseases, whereas monounsaturated fatty acids (MUFA) and polyunsaturated fatty acid (PUFA) are essential to prevent cardiovascular disease (49–53). n-3 PUFA are beneficial to human health and can reduce the incidence of fatty liver, cardiovascular disease and arthritis (54–56). The ratio of PUFAs, n-6 PUFAs, and n-3 PUFAs is widely used as an important parameter to evaluate the nutritional value of meat. The suggested PUFA/SFA ratio is ≥0.4, and the suggested n-6/n-3 ratio is ≤4:1 (57). In our research, the PUFA/SFA ratio of the four mutton breeds ranged from 0.21 to 0.35, which is lower than the recommended range, and the n-6/n-3 ratio ranged from 5.88 to 11.09, which is higher than the recommended range (Supplementary Table 4). These data suggest that mutton is not a good source of dietary fatty acids. Ruminants have lower PUFA/SFA ratios than do non-ruminants because of the biohydrogenation of dietary unsaturated fatty acids by rumen microorganisms (58). Furthermore, C18:2n-6 exists in far lower levels in ruminants than in non-ruminants, which results in a PUFA/SFA ratio well below the recommended value of 0.4 for meat (59). Consequently, we selected the fatty acid indexes of PUFA/SFA and n-6/n-3 to calculate membership function.

### Calculation of the Comprehensive Membership Function Value

In total, we selected 10 indicators for our membership function calculations, of which, six were positive indicators (cooked meat rate, protein, ash, EAA/NEAA, umami amino acid, and PUFA/SFA), and four were negative indicators (water loss rate, shear force, fat, n-6/n-3). Larger positive indexes and smaller negative indexes correspond with better meat quality. Our date showed that the comprehensive membership values of the four breeds in order of highest to lowest were Tibetan sheep (0.76) > Dongxiang tribute sheep (0.49) > Hu sheep (0.46) > Ujumqin sheep (0.33) (Table 1). Mutton quality is difficult to evaluate using a single index alone. If the difference is large, the absolute value of the indicator will take up a large proportion of the entire evaluation process and will have a greater impact on the evaluation results. Likewise, if the difference is small, the absolute value of this index may contribute little to the comprehensive evaluation process. Correlations between traits and quality can be mistakenly enlarged or reduced. The membership function method in fuzzy mathematics accumulates the membership value of each index of the evaluated breed and obtains a mean number. Larger mean number indicate stronger breed superiority. Our results can be used only as a basis for preliminary evaluations owing to the limited number of breeds and measurement indexes.

**TABLE 1:** Membership function values of mutton quality index in four sheep breeds.

Item		Dongxiang tribute sheep	Tibetan sheep	Ujumqin sheep	Hu sheep
Positive indexes	Cooked meat rate	0.62	1.00	0.00	0.34
	Protein	1.00	0.50	0.00	0.27
	Ash	0.00	0.47	0.11	1.00
	EAA/NEAA	0.00	1.00	0.27	0.61
	Umami amino acid	0.38	0.00	0.77	1.00
	PUFA/SFA	0.43	1.00	0.00	0.36
Negative indexes	Water loss rate	0.00	1.00	1.00	0.93
	Shear force	1.00	0.55	0.00	0.37
	Fat	0.32	1.00	0.40	0.00
	n-6/n-3	1.00	0.92	0.49	0.00
Average positive membership function value		0.41	0.66	0.19	0.60
Average negative membership function value		0.58	0.87	0.47	0.33
Comprehensive membership function value		0.49	0.76	0.33	0.46

*EAA, essential amino acids; NEAA, non-essential amino acids; PUFA, polyunsaturated fatty acid; SFA, saturated fatty acid.*

### Gas Chromatography and Ion Mobility Spectrometry Topographic Map of the Dongxiang Tribute Mutton

**FIGURE 1:**
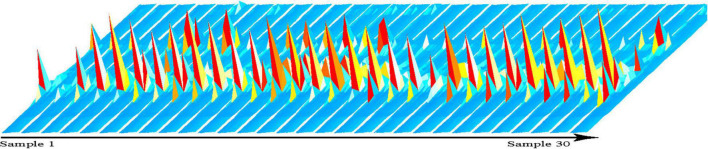
GC-IMS 3D spectrum of part Dongxiang tribute mutton.

**FIGURE 2:**
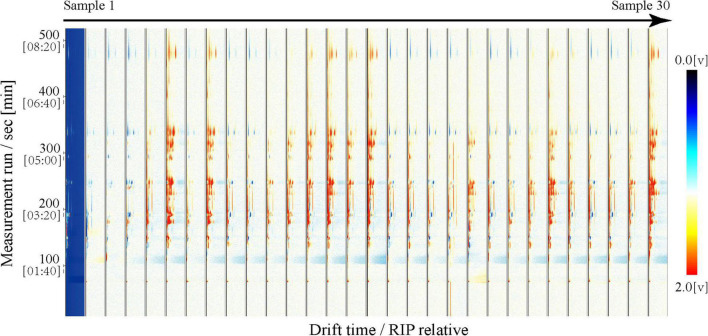
GC-IMS 2D map of Dongxiang tribute mutton.

We used GC-IMS technology to analyze the volatile compounds of mutton from 30 Dongxiang tribute sheep. We used the Receptor plug-in in LAV software to make 3D and 2D comparison images. All 30 samples presented similar RIP signal distributions, indicating highly similar volatile compounds among the individual mutton samples (Figure 1). The RIP intensities did not significantly differ among the samples, indicating that the volatile compound contents in the Dongxiang tribute mutton were relatively stable. To clearly compare the differences in volatile compounds among the 30 samples, a difference comparison model was used to draw a 2D comparison map of the Dongxiang tribute mutton (Figure 2). The 2D comparison draft showed lower signal intensities of certain compounds in samples 2, 3, and 4.

### Identification and Fingerprint Construction of Volatile Compounds in Dongxiang Tribute Sheep Mutton

**FIGURE 3:**
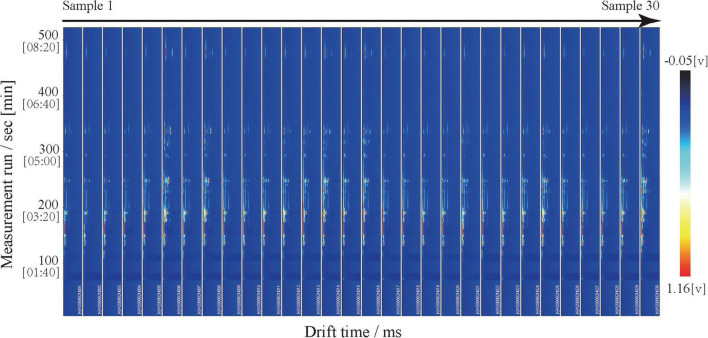
Ion migration spectra of Dongxiang tribute mutton.

Identification of volatile compounds in meat to distinguish between different meat types is another method of food certification (60–62). We qualitatively analyzed the flavor substances using the NIST and IMS databases in the GC-IMS Library Search software And identified 30 volatile compounds: seven alcohols, eight aldehydes, five ketones, two esters, two phenols, one ether, one furan, one acid, two hydrocarbons, and one pyrazine (Figure 3 and Table 2). These volatile compounds contained two dimers because adducts formed between the analyzed ions and neutral molecules (e.g., dimers or trimers), and a single compound can give off multiple signals when passing through the drift zone (63, 64). Of these compounds, the relative content of ketones was the highest, followed by aldehydes and alcohols, which was consistent with results of previous goat meat and beef (65, 66).

**TABLE 2:** Volatile compounds tested in Dongxiang tribute mutton using GC-IMS.

Volatiles	Compound	Average of RIP peak	Retention index	Retention times (s)	Drift times (ms)
Alcohols	1-Octen-3-ol	0.51647	641.6	164.385	1.174
	5-Methyl-2-furanmethanol	0.558384	524.2	111.93	1.125
	2-Phenethanol	1.372805	791.4	273.39	1.57
	(E)-3-hexenol	3.79438	762.2	247.26	1.511
	3-Octanol	0.619748	903.5	403.65	1.332
	1-Octyl alcohol	0.501455	966.8	504.27	1.476
	Phenethyl alcohol	0.461617	675.7	184.275	1.122
Aldehydes	Phenyl propionaldehyde	0.455879	629.6	157.95	1.067
	5-Methylfurfural	2.111457	738.4	227.955	1.537
	Phenyl acetaldehyde	2.167857	857.7	343.98	1.306
	Phenylacetaldehyde*	0.784228	966.6	503.88	1.598
	3-(Methylthio)propionaldehyde	2.116544	966.2	503.1	1.151
	Heptanal	0.18804	763.8	248.625	1.25
	Benzaldehyde	0.520295	829.8	312.195	1.292
	(E)-oct-2-enal	1.068709	907	408.72	1.093
Ketones	Pinocarvone	1.105333	528.5	113.49	1.185
	2,5-Dimethyl-4-hydroxy-3-(2H) furanone	2.141081	715.7	210.99	1.338
	Heptan-2-one	3.806792	492.5	101.205	1.047
	2-Octanone	4.827199	685.8	190.71	1.184
	6-Methyl-5-hepten-2-one	0.823862	1093	788.1899	1.475
Esters	Ethyl octanoate	0.590856	759.2	244.725	1.458
	Methyl caprylate	1.136463	750.9	237.9	1.377
Phenols	4-Methyl guaiacol*	1.141875	925.5	436.02	1.084
	2-Methoxyphenol	0.747482	659.8	174.72	1.162
Ether	Diethylene glycol dimethyl ether	1.193921	906.8	408.33	1.215
Furan	2-Pentylfuran	1.081568	998.1	563.16	1.438
Acid	Pentanoic acid	0.352601	1019.4	607.23	1.404
Hydrocarbons	N-pentylcyclohexane	0.49295	857.3	343.59	1.525
	1,2-Dimethylbenzene	0.93011	999.5	565.89	1.394
Pyrazines	2,6-Dimethylpyrazine	0.335307	778.5	261.495	1.091

*: Dimers formed in the IMS drift tube were represented by symbol “*”; RIP: reactive ion peak.

**FIGURE 4:**
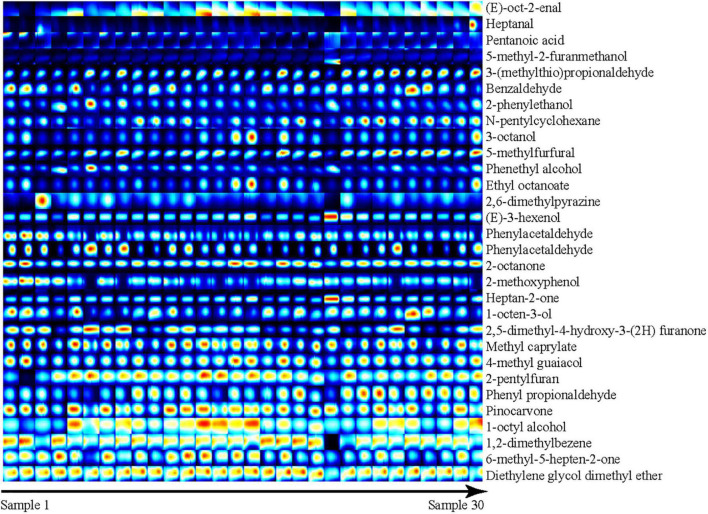
Fingerprint of volatile compounds in Dongxiang tribute mutton.

To more intuitively compare the differences in volatile compounds among individual Dongxiang tribute sheep, we constructed a fingerprint of Dongxiang tribute mutton using the gallery plug-in in LAV software (Figure 4). The volatile compound content in the 30 samples showed few differences, indicating that the Dongxiang tribute mutton samples were relatively pure. Our results also showed that Dongxiang tribute mutton had higher levels of diethylene glycol dimethyl ether, 6-methyl-5-hepten-2-one, 1,2-dimethylbenzene, 1-octyl alcohol, pinocarvone, phenyl acetaldehyde, 2-pentylfuran, 4-methyl guaiacol, methyl caprylate, 2-octanone, and (E)-oct-2-enal and lower levels of heptanal, pentanoic acid, and 5-methyl-2-furanmethanol.

Ketones are the largest volatile compounds in Dongxiang tribute mutton. As the products of lipid oxidation, ketones produce mostly fruity and creamy flavors (67). Additionally, the threshold value of ketones is much higher than that of aldehydes; hence, ketones positively contribute to the volatile flavor of Dongxiang tribute mutton. Heptan-2-one, which has a pear aroma, was detected in Dongxiang tribute mutton. Similarly, Resconi et al. found a significant correlation between heptan-2-one and mutton flavor, indicating that heptan-2-one plays a vital role in changing the volatile flavors of meat products and can be used as a marker of product deterioration (65, 68). Furanones have a gravy aroma and can form at lower temperatures in a shorter time. 2-pentylfuran and other furan compounds are reportedly derived from the Maillard reaction and Strecker degradation of proteins, and the Maillard reaction, lipid thermal degradation and the interaction between these two reactions are the main processes by which flavor and aroma compounds are produced (69, 70).

Aldehydes are the second largest volatile compounds in Dongxiang tribute mutton as well as the main volatile compounds in chicken and pork (71, 72). Aldehydes are the crucial intermediates in the Maillard reaction or lipid oxidation reactions and participate in the interaction between amino acids and carbonyl groups. Aldehydes typically have a relatively low threshold, which is considered to heavily influence the volatile flavor of mutton (73). Most aldehydes have fatty aromas, but high concentrations produce rancidity and other odors (74, 75). Heptanal, which was detected in the Dongxiang tribute mutton, is a key volatile compound in the oxidation process of yak meat and is mainly produced *via* oxidation of linoleic acid (67). Previous studies found that heptanal contents were higher in the volatile compounds of Dorper sheep than in those of Tan and Hu sheep (76). Dorper sheep are thin-tailed sheep, whereas Tan and Hu sheep bare fat-tailed sheep; thus, the fatty acid oxidation process differs between them, and different heptanal contents affect their flavor. Benzaldehyde detected in Dongxiang tribute mutton is a derivative of α-linolenic acid, which has a bitter almond flavor and can negatively affect flavor.

Alcohols are the third most volatile compound in Dongxiang tribute mutton, and their effects on flavor are less significant than those aldehydes (77). Alcohols are produced mainly by oxidation of linoleic acid degradation products, most of which have pleasant odors and can increase the flavor of meat. Mushroom-flavored 1-octen-3-ol was detected in the Dongxiang tribute mutton and other varieties of mutton and contributes greatly to mutton flavor (15, 76). 1-Octen-3-ol was also detected in Dorper, Tan and Hu sheep mutton, and its content was significantly higher in Dorper sheep than in Tan and Hu sheep; thus, it may play an important role in mutton flavors (76). 1-Octen-3-ol is a hydroperoxide degradation product of linoleic acid, and changes in its contents can reflect the racemization degree of meat (78). Although alcohols weakly affect meat flavor, they have a high threshold in sensory analyses and thus synergistically affect the overall smell.

Esters are usually formed by the interaction of alcohols produced by oxidation of free fatty acids and lipids. Among them, short-chain fatty acids have a typical fruity flavor, and long-chain fatty acids have an oily flavor (79, 80). Esters are typically present in limited amounts in meat and contribute little to the flavor. Fewer phenolic compounds are present in meat and are mainly produced *via* pyrolysis of lignin during combustion of fruit trees, such as 4-methyl guaiacol detected in Dongxiang tribute mutton. Previous studies demonstrated that cresol was positively correlated with mutton aroma, but it was undetected in Dongxiang tribute mutton (81). Furthermore, hydrocarbon compounds are mainly produced by homolytic cleavage of fatty acid alkoxide radicals. Owing to their high threshold of flavor, they contribute little to the direct flavor of meat but more to the overall meat flavor (82). Additionally, alkane detection is influenced by the GC-IMS method because the sample and carrier gas both contain water vapor, which makes it difficult to determine the charges of the alkanes (61, 83). Furans are the most abundant volatile products of the Maillard reaction and help coordinate and balance the flavor. The 2-pentylfuran detected in the Dongxiang tribute mutton is an oxidized product of linoleic acid and produces a barbecue flavor (76). The ether content in meat is also limited and contributes little to meat flavor. Pyrazines process unique sensory characteristics that result in many important flavoring agents contents in many fermented and baked foods. Their threshold is lower and relatively stable in the final products of the Maillard reaction, and they contribute to mutton flavor (83). Acids are generated by heating oxidation or enzymatic hydrolysis of fatty acid glycerides and phospholipids. Because short-chain fatty acids are typical odor substances with generally low volatility, they contribute little to the aroma of meat (79).

### Similarity Analysis of Volatile Compounds in Dongxiang Tribute Mutton

**FIGURE 5:**
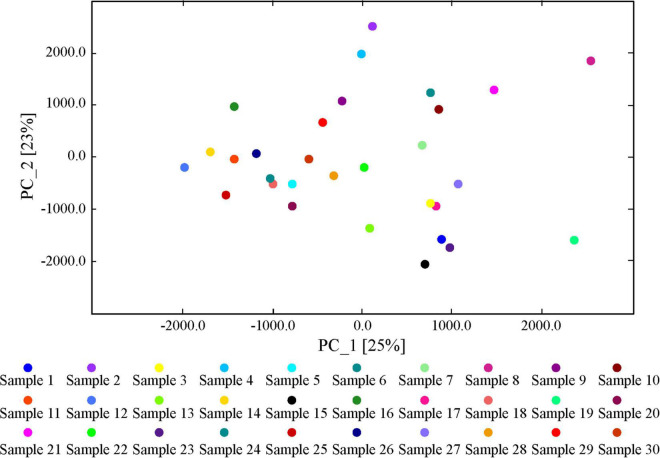
PCA of flavor components of Dongxiang tribute mutton.

**FIGURE 6:**
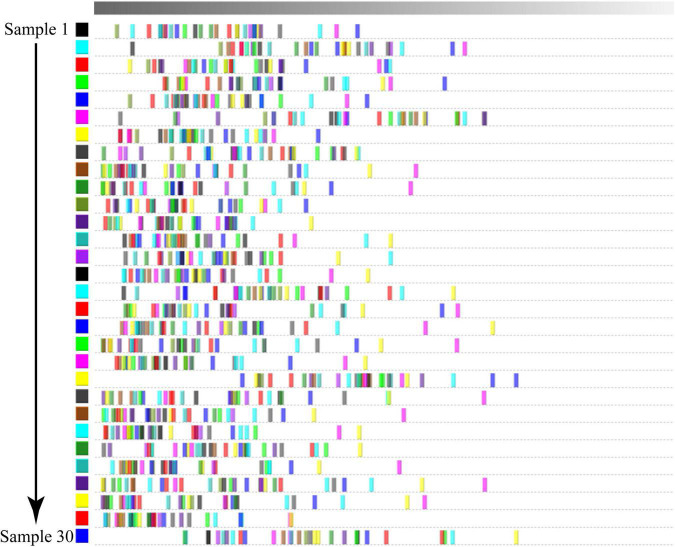
Similarity analysis of volatile components in different Dongxiang tribute mutton.

We conducted a PCA of in the Dongxiang tribute mutton samples using Dynamic PCA plug-ins in LAV software. The contribution rates of principal component (PC)1, PC2, and PC3 were 25%, 23%, and 48%, respectively (Figure 5). Generally, when the cumulative contribution rate reaches 60%, PCA can be used as the separation model. Therefore, the two main components of the 30 Dongxiang tribute mutton samples could not be distinctly separated. We used the matching matrix plug-in in LAV software to analyze the similarities of the volatile components in the Dongxiang tribute mutton. The samples presented > 72% similarity; thus, the sample distribution was concentrated, and the volatile components in the samples were relatively similar (Figure 6). PCA and similarity analysis showed that a similar between the 30 individual samples, with good parallelism; therefore, the results were reliable.

## Conclusion

We calculated the membership function to comprehensively evaluate the quality of Dongxiang tribute mutton and used GC-IMS to identify the main volatile components in the flavor of Dongxiang tribute mutton. The certain advantages in the meat quality of Dongxiang tribute mutton were found, which was indicated by highest contents of protein, EAA, NEAA, and lowest shear force, n-6/n-3 ratio among the four. We screened 10 physical and chemical indicators of meat quality for the membership function calculation, and the meat quality in order of highest to lowest was Tibetan sheep (0.76) > Dongxiang tribute sheep (0.49) > Hu sheep (0.46) > Ujumqin sheep (0.33). Tibetan mutton had the best quality, followed by Dongxiang tribute, Hu and Ujumqin mutton. GC-IMS identified 30 volatile compounds in Dongxiang tribute mutton, and diethylene glycol dimethyl ether, 6-methyl-5-hepten-2-one, 1,2-dimethylbenzene, 1-octyl alcohol, pinocarvone, and 2-phenethanol were the main components contributing to Dongxiang tribute mutton flavor. In this study, we, for the first time, used the membership function method to comprehensively evaluate mutton quality. This method compensated for deviations caused by using a single index alone and yielded more accurate results. GC-IMS enabled identifying the main volatile flavor compounds in Dongxiang tribute mutton. These compounds can be used as markers to identify true and false compounds and expand the practical value of GC-IMS in evaluating meat quality.

## Data Availability Statement

The original contributions presented in the study are included in the article/Supplementary Material, further inquiries can be directed to the corresponding authors.

## Author Contributions

ZL, JBL, YG, and XS conceived and designed the study. JYL, BY, XM, and YY collected the samples. ZL, CY, BX, and TG performed the experiments and analyzed the data. ZL and JYL wrote the manuscript. ZL contributed to revisions of the manuscript. All authors read and approved the manuscript.

## Conflict of Interest

The authors declare that the research was conducted in the absence of any commercial or financial relationships that could be construed as a potential conflict of interest.

## Publisher’s Note

All claims expressed in this article are solely those of the authors and do not necessarily represent those of their affiliated organizations, or those of the publisher, the editors and the reviewers. Any product that may be evaluated in this article, or claim that may be made by its manufacturer, is not guaranteed or endorsed by the publisher.
