# Supplementary material for: Evaluation of Mutton Quality Characteristics of Dongxiang Tribute Sheep Based on Membership Function and Gas Chromatography and Ion Mobility Spectrometry
Source: Front Nutr. 2022 May 6;9:852399. doi: 10.3389/fnut.2022.852399 (PMC9122487; doi:10.3389/fnut.2022.852399)
Supplement: Supplementary file 1 [file Data_Sheet_1.pdf]

Table S1 Results of physical indexes of four breed sheep mutton.

Item	Dongxiang tribute sheep	Tibetan sheep	Ujumqin sheep	Hu sheep
pH	6.40±0.24	6.31±0.07	6.83±0.05	6.78±0.06
L^*	34.29±4.01	31.24±0.84	33.96±1.04	34.60±0.90
a^*	10.14±1.33^{b}	16.04±0.69^{a}	14.09±0.89^{a}	14.52±1.02^{a}
b^*	5.03±1.74	5.02±0.57	5.29±0.53	4.87±0.47
Cooked meat rate (%)	62.48±3.13^{b}	64.74±2.62^{a}	58.76±0.81^{c}	60.78±3.44^{b}
Water loss rate (%)	32.68±3.07^{a}	22.96±1.02^{b}	22.94±1.44^{b}	23.59±1.54^{b}
Shear force (N)	56.26±0.52	58.42±0.48	61.03±0.87	59.25±1.26

Note: Data of mutton quality of Tibetan sheep, Ujumqin sheep and Hu sheep have been published [1], it is only used for the screening of mutton quality indicators for calculation of membership function value. Means within the same row having different superscripts differ significantly ($P < 0.05$), the same as below.

Table S2 Results of routine nutritional components of four breeds sheep mutton.

Item	Dongxiang tribute sheep	Tibetan sheep	Ujumqin sheep	Hu sheep
Water (%)	74.73±1.85	73.31±1.07	76.42±0.31	74.55±0.64
Protein (%)	20.19±1.10	19.45±1.56	18.72±0.65	19.12±0.23
Fat (%)	3.40±0.34^{a}	2.44±0.08^{b}	3.28±0.11^{a}	3.85±0.05^{a}
Ash (%)	1.08±0.05	1.17±0.03	1.10±0.02	1.27±0.04

Table S3 Composition and content of amino acids in mutton of four breeds sheep.

Item	Dongxiang tribute sheep	Tibetan sheep	Ujumqin sheep	Hu sheep
Thr	0.77±0.04	0.68±0.08	0.55±0.05	0.63±0.08
Val	0.90±0.04	1.32±0.12	1.18±0.04	1.28±0.10
Met	0.29±0.04^{c}	0.43±0.08^{b}	0.42±0.04^{b}	0.53±0.05^{a}
Ile	0.76±0.04	0.77±0.10	0.68±0.04	0.72±0.04
Leu	0.76±0.04^{b}	1.38±0.08^{a}	1.30±0.06^{a}	1.28±0.08^{a}
Phe	0.92±0.04^{a}	0.60±0.09^{b}	0.52±0.04^{b}	0.52±0.07^{b}
Lys	1.97±0.09	1.55±0.10	1.37±0.14	1.48±0.08
EAA	7.09	6.73	6.02	6.45
Asp	1.65±0.09	1.33±0.10	1.33±0.05	1.37±0.05
Ser	0.80±0.04	0.78±0.08	0.77±0.08	0.77±0.05
Glu	3.03±0.14	2.82±0.08	2.65±0.14	2.87±0.12
Gly	0.75±0.06	0.77±0.08	0.68±0.04	0.67±0.05
Ala	1.02±0.06^{a}	0.83±0.08^{b}	0.77±0.05^{b}	0.82±0.08^{b}
Cys	0.25±0.03^{a}	0.12±0.04^{b}	0.10±0.00^{b}	0.18±0.04^{a}
Tyr	0.57±0.03	0.48±0.08	0.52±0.04	0.55±0.05
His	0.74±0.07	0.85±0.08	0.75±0.08	0.75±0.05

Arg	1.32±0.06[a]	0.93±0.08[b]	0.93±0.05[b]	0.93±0.05[b]
Pro	0.80±0.07[a]	0.60±0.06[b]	0.57±0.05[b]	0.53±0.05[b]
NEAA	10.94	9.52	9.07	9.43
TAA	18.03	16.25	15.08	15.88
EAA/TAA(%)	39.32	41.41	39.90	40.59
NEAA/TAA(%)	60.68	58.59	60.10	59.42
EAA/NEAA(%)	64.81	70.72	66.40	68.34
Umami amino acid (%)	4.68	4.14	3.98	4.23

Note: EAA, essential amino acids; NEAA, non-essential amino acids; PUFA, polyunsaturated fatty acid; SFA, saturated fatty acid.

Table S4 Composition and content of fatty acids in mutton of four breeds sheep.

Item	Dongxiang tribute sheep	Tibetan sheep	Ujumqin sheep	Hu sheep
C10:0	0.19±0.07	0.11±0.05	0.16±0.02	0.12±0.03
C12:0	0.24±0.04[a]	0.08±0.01[b]	0.16±0.05[a]	0.14±0.05[a]
C13:0	0.1±0.04[a]	0.10±0.04[a]	0.04±0.01[b]	0.04±0.02[b]
C14:0	2.19±0.31	1.44±0.81	2.98±0.59	2.12±0.50
C15:0	0.23±0.09	0.30±0.11	0.29±0.05	0.25±0.08
C16:0	21.96±1.05[a]	14.94±0.91[b]	23.55±1.85[a]	21.11±2.45[a]
C17:0	1.10±0.17	1.10±0.42	0.96±0.02	0.89±0.14
C18:0	21.27±2.21[b]	26.13±1.38[a]	22.03±1.04[b]	21.77±1.50[b]
C20:0	0.19±0.02	0.08±0.03	0.08±0.02	0.10±0.02
C21:0	0.40±0.15[a]	0.28±0.17[a]	0.19±0.04[b]	0.17±0.06[b]
C22:0	0.35±0.08[b]	0.87±0.34[a]	0.62±0.16[a]	0.05±0.01[c]
SFA	48.22	45.45	51.07	46.75
C14:1	0.06±0.01	0.06±0.02	0.07±0.02	0.05±0.01
C15:1	0.35±0.09[a]	0.46±0.15[a]	0.17±0.04[b]	0.12±0.03[b]
C16:1	1.29±0.36	0.87±0.51	1.06±0.19	1.05±0.08
C17:1	0.85±0.12	1.10±0.20	0.87±0.30	0.87±0.18
C18:1n9t	1.98±0.37[a]	2.07±0.79[a]	0.58±0.11[b]	2.44±0.33[a]
C18:1n9c	34.4±1.95	32.22±1.58	34.15±1.75	35.88±0.89
C20:1	0.13±0.08	0.17±0.05	0.28±0.15	0.13±0.02
C22:1n9	-	0.13±0.08	-	-
C24:1	0.44±0.15	0.60±0.26	0.41±0.16	0.42±0.13
MUFA	39.5	37.69	37.58	40.97
C18:2n6t	0.27±0.08	0.27±0.04	0.16±0.11	0.24±0.28
C18:2n6	6.37±1.04	7.65±0.49	5.67±0.83	6.76±0.90
C18:3n3	0.40±0.11[a]	0.43±0.05[a]	0.16±0.02[b]	0.15±0.01[b]
C18:3n6	0.35±0.15	1.09±0.16	0.65±0.25	0.48±0.14
C20:2	0.37±0.09[a]	0.17±0.12[b]	0.12±0.11[b]	0.10±0.03[b]
C20:3n6	0.10±0.04	0.35±0.17	0.09±0.03	0.07±0.01
C20:4n6	3.09±1.15	4.21±1.29	2.67±0.47	3.54±1.38

C20:5n3	1.10±0.18	1.02±0.21	0.60±0.02	0.56±0.09
C22:6n3	0.23±0.07[b]	0.71±0.28[a]	0.32±0.07[b]	0.29±0.05[b]
PUFA	12.28	15.89	10.45	12.19
PUFA/SFA	0.27	0.35	0.21	0.26
n-6/n-3	5.88	6.28	8.56	11.09

References

1. Wang, F.; Wang, H.; Xi, B.; Yang, X.; Li, W.; Gao, Y. Comparison and analysis of meat quality of different breeds of sheep. *Food Ferment. Ind*. **2021**, *47*, 229-235.
